# Evaluation of binding mechanism of dietary phytochemical, capsaicin, with human transferrin: targeting neurodegenerative diseases therapeutics

**DOI:** 10.3389/fphar.2024.1348128

**Published:** 2024-03-01

**Authors:** Mohammed Alrouji, Fahad A. Alhumaydhi, Kumar Venkatesan, Sharaf E. Sharaf, Moyad Shahwan, Anas Shamsi

**Affiliations:** ^1^ Department of Medical Laboratories, College of Applied Medical Sciences, Shaqra University, Shaqra, Saudi Arabia; ^2^ Department of Medical Laboratories, College of Applied Medical Sciences, Qassim University, Buraydah, Saudi Arabia; ^3^ Department of Pharmaceutical Chemistry, College of Pharmacy, King Khalid University, Abha, Saudi Arabia; ^4^ Pharmaceutical Sciences Department, College of Pharmacy, Umm Al-Qura University, Makkah, Saudi Arabia; ^5^ Centre of Medical and Bio-allied Health Sciences Research, Ajman University, Ajman, United Arab Emirates

**Keywords:** neurodegenerative diseases, human transferrin, natural compounds, fluorescence spectroscopy, molecular dynamics simulation

## Abstract

Human transferrin (htf) plays a crucial role in regulating the balance of iron within brain cells; any disruption directly contributes to the development of Neurodegenerative Diseases (NDs) and other related pathologies, especially Alzheimer’s Disease (AD). In recent times, a transition towards natural compounds is evident to treat diseases and this shift is mainly attributed to their broad therapeutic potential along with minimal side effects. Capsaicin, a natural compound abundantly found in red and chili peppers, possess neuroprotective potential. The current work targets to decipher the interaction mechanism of capsaicin with htf using experimental and computational approaches. Molecular docking analysis revealed that capsaicin occupies the iron binding pocket of htf, with good binding affinity. Further, the binding mechanism was investigated atomistically using Molecular dynamic (MD) simulation approach. The results revealed no significant alterations in the structure of htf implying the stability of the complex. *In silico* observations were validated by fluorescence binding assay. Capsaicin binds to htf with a binding constant (*K*) of 3.99 × 10^6^ M^−1^, implying the stability of the htf-capsaicin complex. This study lays a platform for potential applications of capsaicin in treatment of NDs in terms of iron homeostasis.

## Introduction

Alzheimer’s disease (AD) is responsible for 80% of dementia cases, implying the burden this Neurodegenerative disease (ND) is posing in current times (Posner et al., 2002). AD is a progressive, long-term neurological illness, clinically defined by cognitive impairment in several domains, namely, behavior, language, memory, and visuospatial ability (Caballol et al., 2007; Dumurgier and Sabia, 2021). The two major hallmarks associated with onset of AD are accumulation of amyloid beta peptide as senile plaques and pathological hyperphosphorylation of the tau protein, leading to the generation of neurofibrillary tangles (NFTs) (Malafaia et al., 2021; Wegmann et al., 2021). Even though decades of study have not been able to pinpoint the exact etiological causes, it is now understood that AD pathogenesis is intricate and multifaceted.

As of right now, FDA-approved drugs merely temporarily stop the symptoms; they don’t stop the disease’s underlying course. Cholinesterase inhibitors such as donepezil and memantine, as well as NMDA receptor blockers, are approved choices (Calhoun et al., 2018). Lecanemab is a recently approved medication, albeit with some drawbacks. Several studies focusing on tau, amyloid, and other disease pathways try to alter the illness. It is likely that combination therapy will be required due to the complex reasons. In addition to medication-based strategies, lifestyle changes like social engagement, physical activity, and cognitive training may help postpone cognitive deterioration (Williams and Kemper, 2010). Globally, dementia is thought to affect approximately 55 million people, and by 2050, that number is expected to quadruple, posing a serious threat to public health.

Although iron is a necessary mineral, too much of it can cause oxidative stress and brain cell damage (Peng et al., 2021; Zhang et al., 2021). In order to preserve the ideal balance, iron levels are therefore strictly controlled in the brain. Abnormal iron accumulation has been found in the brains of AD patients, particularly among microglia, tau tangles, and amyloid plaques, according to imaging and histology studies. Numerous processes can result in neuronal injury from elevated iron levels. It catalyzes the production of reactive oxygen species, including hydroxyl radicals, which damage proteins, lipids, and genetic material. Additionally, too much iron encourages amyloid-beta to group together to produce harmful oligomers and fibrils that result in plaques (Wang et al., 2019; Onukwufor et al., 2022; Wang et al., 2022). It might also affect abnormal hyperphosphorylation of tau, which would lead to tau misfolding and knotted clusters. In the meantime, normal iron-regulating proteins are disrupted by amyloid and tau clumps, which lead to additional iron imbalances and elevated oxidative stress in a vicious cycle of degeneration (Chen et al., 2017). Excess iron accumulates at the molecular level in mitochondria, interfering with the electron transport chain and preventing cells from producing energy. It also tampers with neurons’ ability to digest lipids. High iron levels in microglial cells cause oxidative damage and toxicity to spread to other neurons by inducing a pro-inflammatory state. Increased neuronal death and the loss of synaptic connections between neurons have been related to dysregulation of iron metabolism (Gleitze et al., 2021). Increased “redox-active iron deposits” in the hippocampus are correlated with declining cognitive function in AD patients, according to neuroimaging data. Restoring equilibrium in iron control and reducing iron-catalyzed oxidative stress may mitigate AD-induced synaptic and neuronal damage. All these reports emphasize the deleterious effects of excessive signifying the importance of keeping a check on iron levels and this is largely maintained by Human transferrin (htf). A glycoprotein that is mostly produced in the liver, htf is essential for maintaining iron homeostasis. It moves iron from places of absorption, storage, and cellular utilization via the circulation. It transports iron to cells without enabling toxicity by binding ferric iron firmly yet reversibly. Although iron is necessary for several essential bodily processes, too much of it can be hazardous. Iron is transported and regulated throughout the body, including the brain, by htf. Elevated iron levels raise the amount of non-htf bound iron and induce htf to saturate, which can result in an iron buildup in tissues (Gosriwatana et al., 1999; Leverence et al., 2010). Within the brain, there are receptors on the endothelial cells of the blood brain barrier that binds htf, thereby controlling iron entry into the brain (DeGregorio-Rocasolano et al., 2019). Further to avoid the toxicity, it also controls iron release from glial cells and neurons (Ward et al., 2014). There is a rare ND wherein iron accumulates in the brain has and it has been connected to mutations in the htf gene reducing the protein’s affinity for iron.

In recent times, many studies have reported that there are number of plant derived substances and dietary components that may be useful in AD therapeutics. Polyphenols are usually found in fruits, vegetables, tea, coffee, and herbs and possess variety of neuroprotective properties, namely, the reduction of amyloid and tau lesions, limiting neuroinflammation, simulating brain plasticity and connections, and reducing oxidative stress. Studies show that populations with high polyphenols consumption shows reduced incidences of AD (Jiang et al., 2013; Ozcan et al., 2014; Uddin et al., 2020).

Capsicum genus plants contain capsaicin (8-methyl-N-vanillyl-6-nonenamide), a hydrophobic, spicy-tasting transient receptor potential vanilloid 1 (TRPV1)-receptor agonist (Chang et al., 2011; Gonzalez-Mondragon and Vazquez-Tzompantzi, 2011). People frequently use capsicum in their meals due to its flavor and spice. The daily intake of capsaicin is estimated to range from 1.5 mg per person in the US and Europe to 25 mg per person in India and 200 mg per person in Mexico (Rollyson et al., 2014). According to animal studies, 50%–90% of capsaicin is available orally. Capsaicin passes the blood-brain barrier, as demonstrated by animal research, which is necessary for it to be taken into consideration for AD treatment. From a physiological perspective, capsaicin is well known for its capacity to elicit pain and sensitize peripheral and central nerves, resulting in symptoms that resemble neuropathic pain, including visceral hyperalgesia, secondary hyperalgesia, transferred pain region, and allodynia.

Capsaicin is widely used as a topical analgesic (Fattori et al., 2016), neuro-protectant, reduces oxidative stress and enhances apoptosis in epilepsy and ischemic injury (Khatibi et al., 2011; Abdel-Salam et al., 2020). It also reduces inflammation (Tang et al., 2015; Shang et al., 2017) and exhibits antioxidant properties (Galano and Martínez, 2012; Lu et al., 2020). Capsaicin has been demonstrated recently to reduce tau hyperphosphorylation in rats with Type 2 diabetes (T2D) after they received an injection of a streptozocin AD model (Xu et al., 2017). Spicy food consumption has been linked to improved cognitive function in humans, and in non-AD subjects, reduces CSF phospho-tau/Aβ1-42 and total tau/Aβ1-42 ratios have been reported (Liu et al., 2016; Tian et al., 2021). All these reports signify the importance of capsaicin in NDs therapeutics. Thus, *in lieu* of the importance of htf and capsaicin in NDs therapeutics, the current work targets to decipher the binding mechanism of capsaicin with htf through computational and experimental approaches. The binding of capsaicin with htf was delineated at atomistic level using molecular docking and molecular dynamic simulation approaches and further validated by fluorescence spectroscopic technique.

## Materials and methods

### Materials

htf and capsaicin were purchased from Sigma Aldrich (St. Loius, United States). We prepared a stock solution of htf and it was subjected to further dilution for working concentrations. All the chemicals used for buffer preparation were obtained from HiMedia. We used quartz cuvettes for spectroscopic assays.

### Molecular docking

Molecular docking was employed to explore the binding affinity and interaction between htf and capsaicin. The structure of htf (PDB accession: 3V83) was extracted from the RCSB Protein Data Bank with. The ligand structure was retrieved from the PubChem database with CID: 1548943. The structures were processed utilizing MGL AutoDock tools (Huey et al., 2012). Molecular docking was carried out using InstaDock v1.2 to investigate detailed binding of capsaicin with htf. Htf (Mohammad et al., 2021). We used a blind search space strategy to ensure thorough exploration of potential binding sites within the protein. was employed. For the experiment, the grid coordinates were set at 82 Å for the X-axis, 99 Å for the Y-axis, and 84 Å for the Z-axis, with the central coordinates defined as follows: −52.355 along the X-axis, 17.601 along the Y-axis, and −30.21 along the Z-axis. We used a grid spacing of 1 Å and retorted employed Discovery Studio Visualizer and PyMol to carry out post docking visualization.

### MD simulations

Molecular dynamic (MD) simulation studies were performed under a constant temperature of 300 K, using the “charmm36-jul2022” force field within the “GROMACS suite version 2020-β” (Van Der Spoel et al., 2005). The best pose that was obtained from docking studies was used as a starting structure. Ligand topology was created through the CHARMM CGenFF program. Subsequently, the integration of the ligand topology with the initial coordinates of htf was carried out using CGenFF Python script to ensure proper configuration of the system. After that the solvent molecules were introduced in the system by utilizing a 1 nm cubic box and the solvation was executed using the *gmx solvate* command, employing SPC216 solvent in GROMACS (Glättli et al., 2002). Post that energy minimization was carried out using the steepest descent approach to refine the systems. The resulting trajectories were investigated using various GROMACS modules, including *gmx rms, gmx rmsf, gmx gyrate, gmx sasa, gmx sham*, etc. The graphs and visual representations were generated using QtGrace (Turner, 2005).

### Fluorescence spectroscopy

We employed fluorescence binding study to check the actual binding of capsaicin with htf in line with earlier studies on Shimadzu Spectrofluorophotometer (RF-6000). The concentration of htf was kept constant (4 µM) and capsaicin was varied in the range of 0–6 µM. The data obtained was put into Modified Stern–Volmer (MSV) equation as per earlier literature to obtain the binding constant (*K*) of Htf-capsaicin complex (Shamsi et al., 2020). We carried out the experiment in triplicates and mean value was taken into account. All the spectra reported here are the subtracted spectra after considering the fluorescence of capsaicin by taking a blank spectra of the ligand.

## Results

### Molecular docking

**FIGURE 1:**
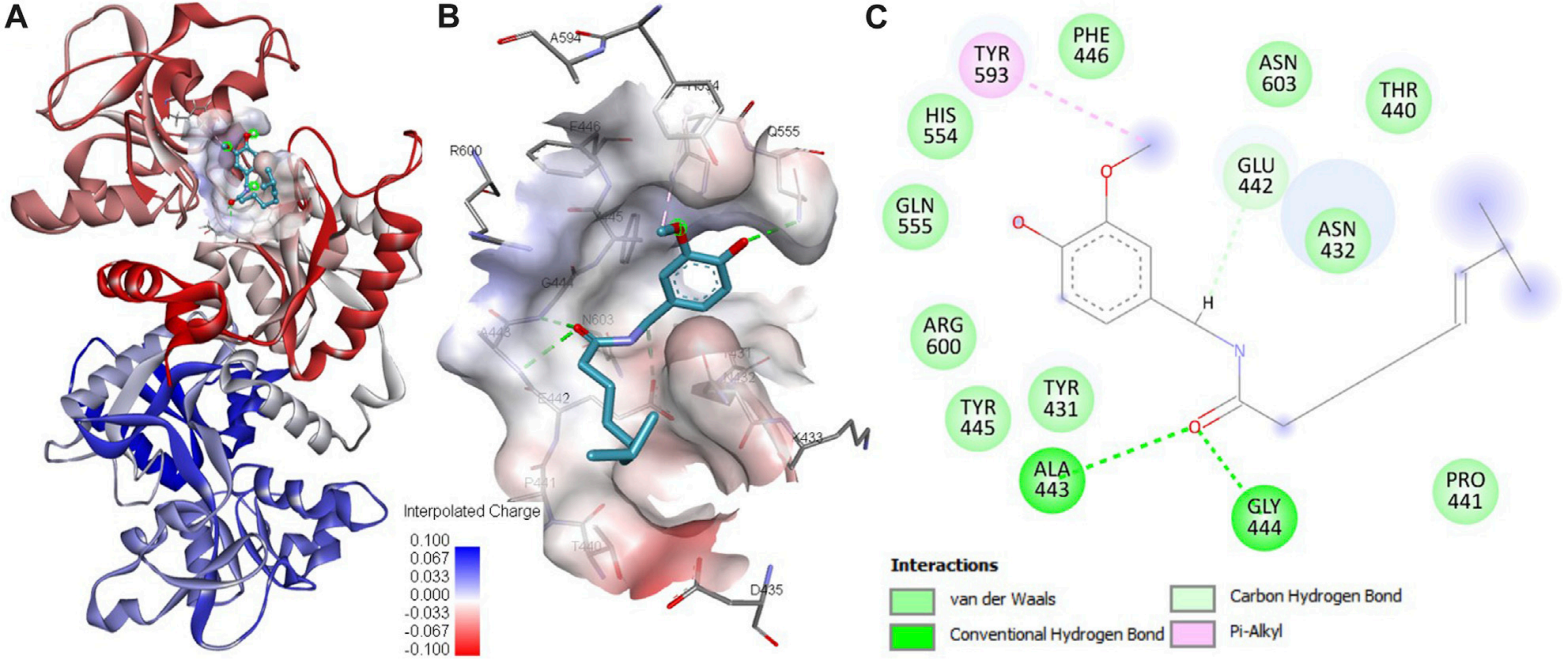
Analysis of protein-ligand interactions. **(A)** Binding of capsaicin with htf. **(B)** Depiction of capsaicin docking and its interaction with the binding site residues of htf. **(C)** Visualization of the surface potential within the htf binding pocket, featuring capsaicin occupancy.

The outcomes derived from our molecular docking study have uncovered a robust interaction between capsaicin and htf, emphasizing it to be a potential binding partner. This docking analysis has furnished better comprehension of the binding mode of capsaicin with htf, allowing for the recognition of critical residues involved in this interaction. The computed affinity for the htf-capsaicin was found to be −5.9 kcal/mol, accompanied by a ligand efficiency of 0.2682 kcal/mol per non-hydrogen atom. Building upon the observed binding affinity, we conducted an exhaustive analysis to explore the potential interactions stabilizing the htf-capsaicin complex. This analysis has provided a clear understanding of capsaicin’s binding mechanism against htf, highlighting its preference for occupying htf’s iron-binding pocket, strategically positioned within the deep cavity of htf (Figure 1A). This positioning establishes significant connections with residues located in the binding pocket, as depicted in Figure 1B. Notably, three hydrogen bonds are formed with Glu442, Ala443, and Gly444, crucial residues in close proximity to htf’s iron-binding site (Noinaj et al., 2012). Visual representations of the surface further accentuate capsaicin’s presence within htf’s internal cavity, as shown in Figure 1B. Moreover, a range of significant interactions, including the iron-binding site Tyr445, contributes to the stability of the protein-ligand complex, as illustrated in Figure 1C. Earlier studies have similarly reported binding of iron in vicinity of these crucial residues (Noinaj et al., 2012). Therefore, a reasonable hypothesis can be proposed that capsaicin docks in close proximity to htf’s iron-binding pocket, establishing stable interactions with various binding site residues within htf.

### Htf dynamics upon capsaicin binding

**FIGURE 2:**
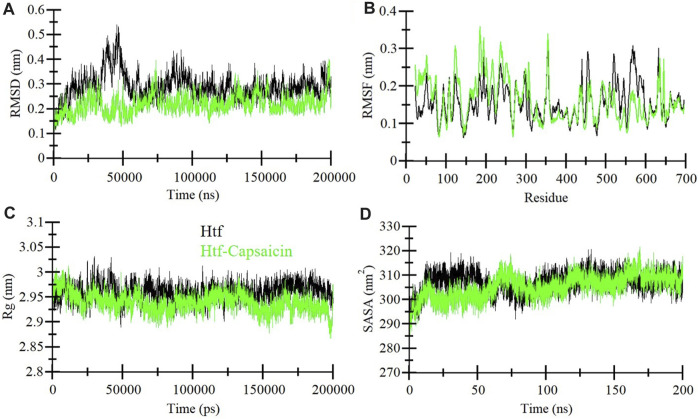
Dynamics of htf structure in the presence of capsaicin. **(A)** Root mean square deviation (RMSD), **(B)** Root mean square fluctuation (RMSF), **(C)** Radius of gyration (*R*g), and **(D)** Solvent accessible surface area (SASA) plots. The black and green correspond to the values obtained for free htf and htf-Capsaicin complexes, respectively.

MD simulation is extensively utilized to explore the dynamic motions and conformational changes of macromolecules over a defined time frame. Here, we performed thorough MD simulations, spanning a duration of 200 ns, for htf and htf-capsaicin complex. Our primary aim was to explore conformational alterations, system stability, and the dynamics of interaction involved in the binding of capsaicin with htf. To assess structural dynamics, we employed the commonly used metric known as the root mean square deviation (RMSD) (Maruyama et al., 2023). The RMSD values for htf and htf-capsaicin complex did not reveal significant differences, suggesting that the capsaicin binding stabilizes htf without affecting its native conformation (Figure 2A). However, there were some initial fluctuations in RMSD in the first frame of 50 ns. The observed fluctuations can reasonably be attributed to the initial orientation of capsaicin within htf’s binding pocket. Subsequent to this initial phase, the RMSD of the htf-capsaicin complex stabilizes, emphasizing the sustained stability of the protein-ligand complex (Figure 2A).

We assessed local structural characteristics and the flexibility of each residue in the protein structure by conducting root-mean-square fluctuation (RMSF) calculations for free htf and capsaicin bound htf its (Figure 2B). The obtained RMSF plots show that there are variations in residue flexibility across htf. RMSF of htf persists fairly constant upon the binding of capsaicin, in a way showing intermittent fluctuations and stabilization peaks throughout the structure. Upon capsaicin binding, there was an elevation in residue fluctuations, specifically in the “N-terminal region,” where capsaicin is not bound. The occurrence of this phenomenon is attributable to the residual vibrations persisting throughout the course of simulation.

The radius of gyration (*R*_g_) is a vital parameter that demonstrates protein’s conformational structure (Lobanov et al., 2008). The average *R*_g_ for htf and the htf-capsaicin complex was between 2.9 nm and 3.0 nm. The value of *R*_g_ for the htf-capsaicin complex was slightly lower in comparison to free htf and this was constant throughout the simulation (Figure 2C). The *R*_g_ plots depicted modest shifts with no major structural rearrangements in htf post capsaicin binding. This implies that binding of capsaicin induced higher stability in the native conformation of htf.

Additionally, Solvent-accessible surface area (SASA) was calculated for protein alone and in complex with ligand. SASA sheds light on the extent of a protein’s surface engagement with the surrounding solvent environment (Marsh and Teichmann, 2011). The calculated SASA values for protein alone and in complex with htf bound capsaicin ranged from 295 nm^2^ to 315 nm^2^, respectively. A subtle reduction in SASA was observed implying that there is a degree of conformational stability in htf upon capsaicin binding (Figure 2D). This marginal decline in SASA implies conformational stability in the htf resulting from capsaicin binding, i.e., ligand occupies some intramolecular space of the protein.

### Stabilization of htf-capsaicin complex

**FIGURE 3:**
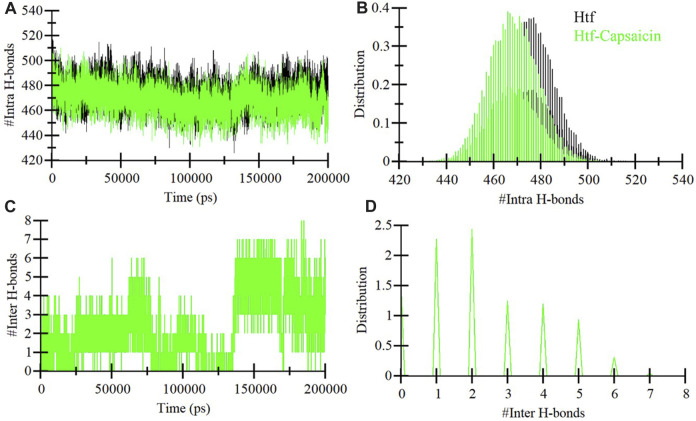
Dynamics of hydrogen bond formation. **(A)** Intra-htf hydrogen bonds, and **(B)** Intermolecular hydrogen bonds between capsaicin and htf.

Integral hydrogen bonding plays a pivotal role in maintaining protein stability and can introduce directionality to interactions between proteins and ligands (Yunta, 2017). In the context of this study, we conducted an exhaustive examination of the stability of htf and htf-capsaicin complex. This investigation encompassed the quantification of hydrogen bonds formed within the protein structure throughout the simulation duration, as depicted in Figure 3A. Significantly, a subtle reduction in the number of hydrogen bonds becomes apparent upon the binding of capsaicin. In contrast, htf and capsaicin consistently maintaining 2–3 intermolecular hydrogen bonds throughout the simulation, as depicted in Figure 3B. Within htf’s binding pocket, the interaction with capsaicin was noted to involve 7–8 hydrogen bonds with increased fluctuations and 2–3 hydrogen bonds up to 130 ns, followed by 4–6 hydrogen bonds after 130–200 ns, demonstrating relatively enhanced stability. A detailed examination of hydrogen bonding patterns facilitates a deeper understanding of the stability of the interaction within the htf-capsaicin complex.

### Principal component analysis

**FIGURE 4:**
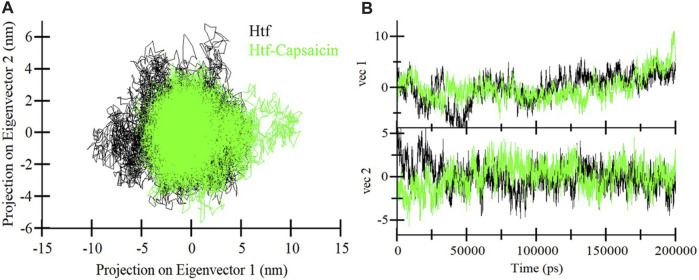
**(A)** 2D representations of htf conformations and **(B)** the temporal evolution of these conformations.

Proteins function through intricate atomic movements, and employing principal component analysis (PCA) offers a valuable approach to unraveling fundamental dynamics by pinpointing key principal motions (Stein et al., 2006). This approach has the capacity to illuminate insights into protein stability. In our exploration of the dynamic conformational behaviors of htf and the htf-capsaicin complex, we employed PCA and visually illustrated the changes in the conformation within the essential subspace (Figures 4A, B). Interestingly, with the introduction of capsaicin, htf demonstrates a cluster of stable states, suggesting a minor reduction in conformational exploration compared to its unbound state (Figure 4B). Overall, the results indicated that htf remained stable before and after capsaicin binding during the simulation trajectory.

### Free energy landscape analysis

**FIGURE 5:**
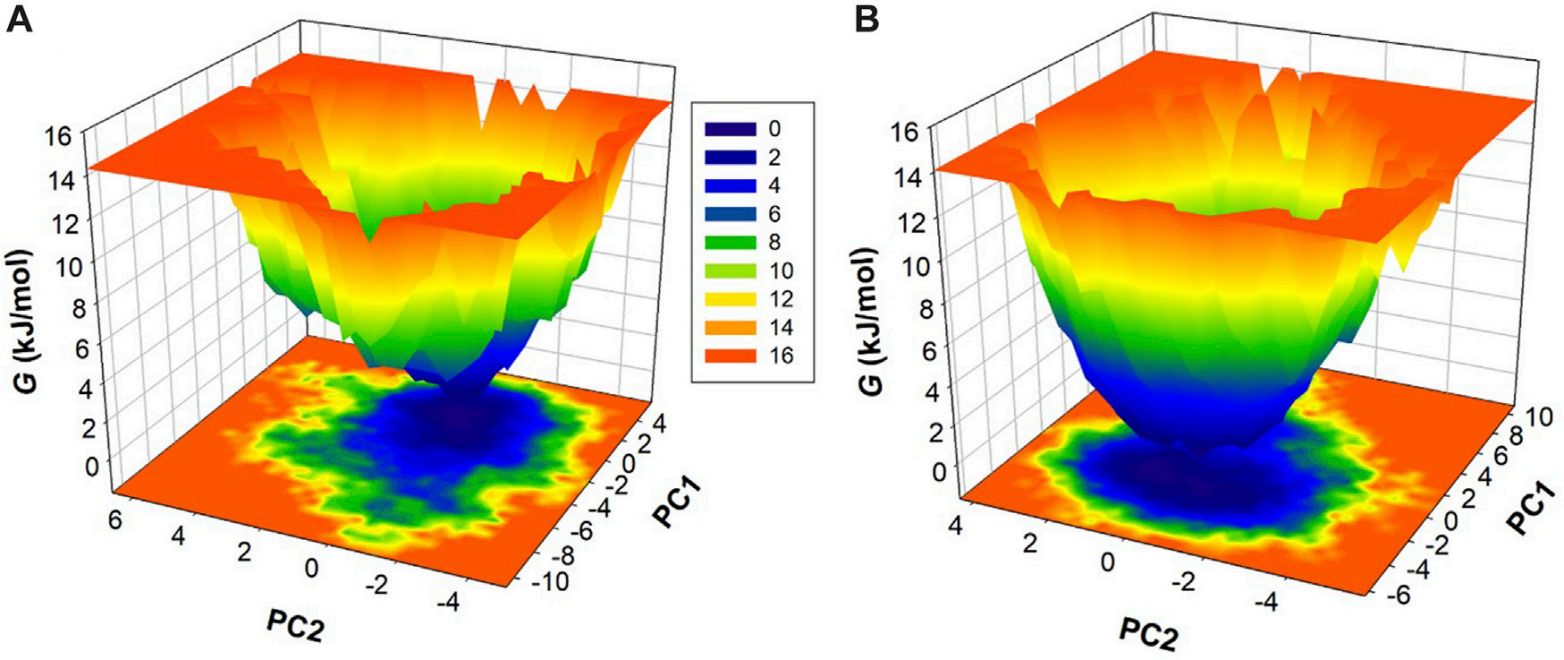
Gibbs’ Free energy landscapes for **(A)** htf and **(B)** htf-capsaicin.

To enhance our understanding of conformational dynamics, we conducted an in-depth exploration of the free energy landscapes (FELs) derived from the MD trajectories. Figure 5 vividly illustrates the FELs for htf and htf-capsaicin complex. In the unbound state of htf, the FEL shows a singular and consistently stable global minimum, primarily confined within a large single basin (Figure 5A). This suggests a well-defined and stable conformational state for htf when not bound to capsaicin. Upon the introduction of capsaicin, the overall conformational behavior of htf undergoes examination (Figure 5B). Strikingly, there is no significant alteration observed, indicating that capsaicin binding does not induce substantial changes in the conformational landscape of htf. The presence of capsaicin, as depicted in the FELs, expands the spectrum of conformational space discovered by htf, suggesting potential influences on its functional dynamics. This stability is crucial in understanding the molecular intricacies governing the interaction between htf and capsaicin and its potential implications for functional dynamics.

### Fluorescence spectroscopy

**FIGURE 6:**
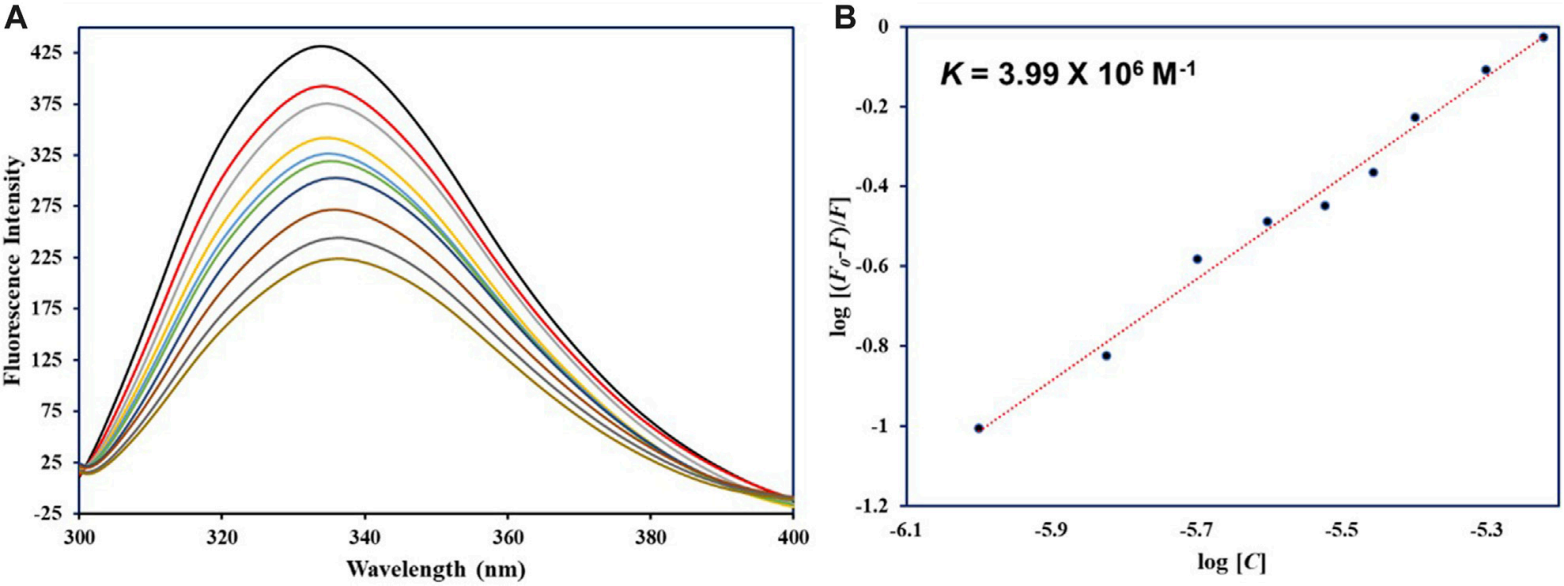
**(A)** Fluorescence emission spectra of native htf in absence and presence of capsaicin. **(B)** MSV plot of htf-capsaicin complex.

Fluorescence spectroscopy ascertains the actual binding affinity of ligand with the protein. For htf, tryptophan alone is main player in the intrinsic fluorescence; tyrosine’s fluorescence undergoes complete quenching when subjected to ionization or when in proximity to an “amino group,” a “carboxyl group,” or another tryptophan residue; phenylalanine has the lowest quantum yield. *In silico* observations showed the formation of stable htf-capsaicin complex. In a bid to further validate these computational observations, fluorescence binding studies were carried out. Intrinsic fluorescence of htf is very responsive to its micro-environment (Sarzehi and Chamani, 2010); any minimal changes in the microenvironment around its fluorophores results in corresponding changes in the fluorescence spectra. We excited htf at 280 nm, and it was found to show characteristic peak at around 335 nm, suggestive of the fact that htf is in its native form. The protein’s fluorescence intensity might decrease due to inner filter effect, which happens when the solution absorbs light at the excitation (λ_ex_) and emission (λ_em_) wavelengths and thus “inner filter’s effect” on the fluorescence intensity was adjusted as per earlier reported studies (Chi and Liu, 2011). With increasing concentrations of capsaicin, a corresponding decrease in the fluorescence intensity was observed, a phenomenon referred to as fluorescence quenching (Figure 6A). This dose dependent decrease fluorescence intensity in the presence of capsaicin is suggestive of the formation of htf-capsaicin complex, validating our *in silico* observations. We fitted the quenching data into MSV equation to obtain MSV plot (Figure 6B) with its intercept giving the binding constant of the complex. Capsaicin demonstrated significant binding with htf with a *K* of 3.99 × 10^6^ M^−1^. The computed *K* value fall within the reported range for other protein-ligand complexes (Alhumaydhi et al., 2021; Anwar et al., 2021), indicating a substantial strength of interaction between capsaicin and htf. In conclusion, fluorescence spectroscopy along with molecular docking and molecular dynamics simulation approaches affirms the fact that capsaicin binds to htf with a significant affinity forming a stable htf-capsaicin complex.

## Discussion

Our comprehensive investigation harnessed an integrated approach utilizing docking and MD simulation along with fluorescence spectroscopy, to decipher the binding of capsaicin with htf. The robust interaction between capsaicin and htf, characterized by binding affinity of −5.9 kcal/mol, firmly underscored capsaicin’s potential as htf’s binding partner. An in depth analysis revealed the key residues governing this interaction; capsaicin forms three hydrogen bonds with critical residues Glu442, Ala443, and Gly444, in close proximity to htf’s iron-binding site. This finding hints at a potential impact of capsaicin’s binding on iron coordination and transport, providing a nuanced understanding of the molecular mechanisms at play.

Subsequent MD simulations spanning htf and htf-capsaicin complex over 200 ns illuminated stability and conformational dynamics. The RMSD analysis indicated stable and sustained conformational changes in htf upon capsaicin binding, reinforcing the notion of complex stability. Notably, heightened fluctuations in residues, particularly within the N-terminal region, suggested localized flexibility introduced by capsaicin binding while preserving overall stability. The *R*g and SASA study provided additional insights into the conformational changes triggered by capsaicin binding. The minimal alterations in overall protein conformation, indicated by a slight increase in *R*g values, further supported the notion of stability. Minor fluctuations in hydrogen bond formation underscored the dynamic nature of the complex, emphasizing the critical role of hydrogen bonds in sustaining stability. PCA indicated that capsaicin binding expanded the range of conformational states explored by htf, suggesting a broader conformational space. The FEL analyses further supported the stability in the conformational behavior of htf initiated by capsaicin binding. Further, fluorescence spectroscopic observations corroborate with *in silico* observations affirming the formation of stable htf-capsaicin complex. In summary, our study has provided a comprehensive analysis of the htf-capsaicin complex, shedding light on its binding affinity, stability, and conformational dynamics. Capsaicin has been identified as a potent htf binding partner, displaying a robust interaction within the iron-binding pocket. The study further elucidates the complex’s stability through a detailed examination of the intricate hydrogen bonding network. The findings from this investigation establish a solid groundwork for future investigations into capsaicin’s therapeutic potential to treat NDs in context of iron proteins. This research contributes significantly to our understanding of the nuanced interplay between htf and capsaicin, offering valuable insights for potential therapeutic applications.

## Data Availability

The original contributions presented in the study are included in the article/supplementary material, further inquiries can be directed to the corresponding author.
